# Modeling spatio-temporal dynamics of network damage and network recovery

**DOI:** 10.3389/fncom.2015.00130

**Published:** 2015-10-22

**Authors:** Mohammadkarim Saeedghalati, Abdolhosein Abbassian

**Affiliations:** BioMath, School of Mathematics, Institute for Research in Fundamental Sciences (IPM)Tehran, Iran

**Keywords:** spatiotemporal dynamics, brain damage, network dynamics, network damage, brain network models, network recovery, fast dynamics, slow dynamics

## Abstract

How networks endure damage is a central issue in neural network research. In this paper, we study the slow and fast dynamics of network damage and compare the results for two simple but very different models of recurrent and feed forward neural network. What we find is that a slower degree of network damage leads to a better chance of recovery in both types of network architecture. This is in accord with many experimental findings on the damage inflicted by strokes and by slowly growing tumors. Here, based on simulation results, we explain the seemingly paradoxical observation that disability caused by lesions, affecting large portions of tissue, may be less severe than the disability caused by smaller lesions, depending on the speed of lesion growth.

## 1. Introduction

The performance of networks enduring damage is a central issue in biological and non-biological networks (Cohen et al., 2000b; Nawrocki and Voyles, 2011). Intuitively, the degree of disability following damage must be correlated with the size of damage. However, different networks architectures, show different responses to spatial pattern of damage (Callaway et al., 2000; Cohen et al., 2000a; Albert et al., 2000; Vazquez and Moreno, 2002; Murre et al., 2003; Bondarenko, 2005; Fortney et al., 2007; Alstott et al., 2009; Lee et al., 2011; Wang et al., 2011).

Attractor neural networks, for example, are known to preserve stored information in spite of big damages incurred on their synaptic connections (Amit, 1992). Moreover, experiments show that it is easier to recover from lesions that grow slowly than from those that grow fast. In fact, recovery from a lesion with given size is possible depending on whether the lesion evolved slowly or fast (Duffau et al., 2002, 2003; Desmurget et al., 2007; Varona, 2010). This is important because in a realistic setting of optimal recovery, the temporal rate as well as the size of injury must be taken into account. Experimental data or rats, cats, and monkeys show a time interval between brain lesions has a strong effect on deficits that animals bear after operation (Stewart and Ades, 1951; Meyer et al., 1958; Adametz, 1959; Finger et al., 1971; Rosen et al., 1971; Glick and Zimmerberg, 1972; Patrissi and Stein, 1975). In a more clinical setting this proves to be a crucial aspect of how the brain recovers from strokes or lesions caused by slow-growing tumors. The biological basis of recovery after stroke and how the brain reorganizes itself, for example, is still largely unknown (Pantano et al., 2003; Calautti and Baron, 2003) but certainly it is possible that the degree and speed of recovery varies considerably for different lesion locations and depends on structural alterations taking place in the spared brain tissue during a slow and gradual temporal lesion (Stewart and Ades, 1951; Meyer et al., 1958; Glick and Zimmerberg, 1972).

In these cases, two main points need to be emphasized. First, the spatial damages are not spread out at random but are highly localized. Second, damages evolve in the face of brain on-going activity. This makes the modeling study of brain damage a challenging task and indeed most of the experimentally observed cases of brain re-mapping remain unexplored in simulation studies. To name a few, there seems to be a clear difference of functional re-mapping to the adjacent lesion areas in slow vs. fast growing tumors (Desmurget et al., 2007). Here, a gradual injury with slow dynamics and with the benefit of brain on-going activity, may increase the healing potential of brain injuries whereas an acute destruction will limit the chance of recovery due to a sudden seize of activity in the damaged brain areas. Neurons near the inflicted areas need time to rewire in order to compensate for the computational share of neurons in the inflicted area. Given a sufficient window of time there is a chance for recovery as long as the resulting disability does not pass a certain threshold. Now, not much is known about the specifics of network damage in the case of brain diseases. In a highly nonlinear dynamics of brain damage, the interplay between an acceptable level of damage and brain ongoing recovery is an important question in clinical studies. As a first step one would want to know how destruction of a given area, changes the pattern of recovery depending on the dynamics of the injury.

Recently, there has been some attempts using simple recurrent networks to show the effect of brain remapping and how it contributes to neuronal homeostasis, following lesions (Butz et al., 2008, 2009). These findings, however, relate more to the spatial pattern of brain lesions and need to be extended to what may be called a spatio-temporal pattern of brain injury. Here, a distinction has to be made between an acute stroke where there is a sudden damage inflicted to the brain and a slow growing lesion such as a low-grade gliomas (Duffau et al., 2002, 2003; Desmurget et al., 2007; Varona, 2010). Based on many experimental reports, the slow growing injuries have a much better chance of being recovered than the injuries caused by acute lesions (Stewart and Ades, 1951; Meyer et al., 1958; Adametz, 1959; Finger et al., 1971; Rosen et al., 1971; Glick and Zimmerberg, 1972; Patrissi and Stein, 1975).

In the present paper two simple but different networks, a three-layer and a homeostasis model with different temporal patterns of damages are studied. These models are not only simple but general enough to account for both biological and non-biological networks. In the former case, for example, a feed-forward network is often considered as appropriate for modeling a sensory processing stage whereas a recurrent network such as the homeostasis model is related to a higher level stage. This, however, is rather arbitrary as it is now possible to design a recurrent network with a feed-forward type of behavior (Goldman, 2009). Still, studying these prototype examples separately is preferred when the underlying mechanism for some common behavior remains unknown.

The three-layer model contains the main ingredients of our network, consisting of an input, a processing and an output layer. Processing layer is where we introduce injuries. Network disability is measured as the Hamming distance between current and the desired output.

The homeostasis model (Butz et al., 2008, 2009) is a more realistic model of a cortical circuit. It was first developed by Butz et al. (2008, 2009) and is based on a recurrent network. For this model the variance of deviation from homeostasis is measured as network disability. Of course, at this level of generality the models are not intended to be any more realistic than being able to show basic trends and functional behavior of the network. The parameters chosen in both models make it possible to study most clearly the effect of damage size relative to the given measures. As will be shown, a gradual injury is indeed resisted by these networks provided the lesions inflicted on the networks do not exceed a critical size. Our main finding is that a gradual injury will decrease the maximum amount of disability, so network may have more chance of recovery. Obtaining the same results using two different models suggests that the results are robust and independent of details of the models.

## 2. Three layer model

### 2.1. The model

In this model neurons of the input layer are connected to the middle (processing) layer with weighted connections. After the processing is done in the middle layer, their activity passes through another set of weighted connections to the output layer. Connections from each layer to the next are locally dense, i.e., each node is connected to a group of neighboring nodes, similar to what is generally observed in the real brain.

The network is expected to produce the desired output based on the input fed to the first layer. Here, the disability of the system is measured by measuring the error ratio in the output layer using Hamming distance. The resulting disability which is caused by a specific pattern of injury inflicted on the middle layer, is studied.

**Figure 1:**
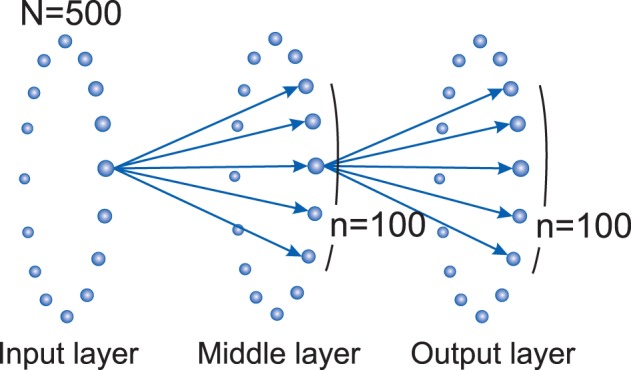
**Three layer model network topology: there are feed-forward connections from each layer to the next one**. Each node from input layer (*I*_*i*_) connects to *n* neighbor nodes in the processing or middle layer ($M_{i-\frac{n}{2}}$ to $M_{i+\frac{n}{2}}$). Same connections exist between middle layer and the output layer. There isn't any connection between nodes of a layer. In this study, we set *N* = 500 and *n* = 100.

In this model, the nodes are binary neurons with two states of 0 and 1, showing inactive and active states, respectively. The network consists of three rings of nodes, each containing *N* nodes. There is no connection between nodes in each layer but there are feed-forward connections from the previous to the next layer. The nodes in input, middle, and output layer are labeled as *I*_*i*_, *M*_*i*_, and *O*_*i*_, respectively, where *i* = 0, 1, 2, …, *N*. Each node *i* is connected to a group of *n* neighboring nodes in the next layer, ranging from $i-\frac{n}{2}$ to $i+\frac{n}{2}$. (see Figure 1).

The activation probability of each node will be shown by its activation function. The activation function of node, *i*, of middle layer at time *t*, is

(1) $$\begin{array}{l} F\left(X_{i}^{t}\right)=\frac{1}{1+e^{-\beta \left(X_{i}^{t}-\theta_{ml}\right)}} \end{array}$$

where θ_*ml*_ is threshold, β is noise amplitude and $X_{i}^{t}=\overset{N}{\underset{j=1}{\sum }}w_{ij}I_{j}$ where *w*_*ij*_ is the weight of connection from the node *j* in the input layer to the node *i* in the middle layer. Nodes in the output layer are binary threshold units with a Heaviside activation function and threshold θ_*output*_. In our model a Hebb-like learning rule is applied and the change in connection weight Δ*w*_*ij*_ between nodes *i* and *j* is:

(2) $$\begin{array}{l} \Delta w_{ij}=\eta \left(as_{i}s_{j}-bs_{i}-cs_{j}\right) \end{array}$$

where *a, b*, and *c* are constants and η is the learning rate, *s*_*i*_ and *s*_*j*_ show the states of the nodes *i* and *j* which are “1” if they are active and “0” otherwise. The constants *a, b*, and *c* are chosen in a way that if two nodes are active at the same time their connection weight increases by η∕2 and decreases by the same amount if only one of the two is active. The weight of the connections is bounded and not allowed to increase or decrease any further. The minimum value is set to zero to prevent a neuron to be both excitatory and inhibitory. The upper bound prevents the system from having infinite connection weights. This upper bound is coupled with the firing threshold of the neuron's activation functions. We use Hamming distance as a measure of the dissimilarity between the actual and the desired output:

(3) $$H=\sum_{1}^{N}|s_{i_{actual\;output}}-s_{i_{desired\;output}}|$$

which counts the number of mismatches between the actual and desired patterns. In this study all the constants are set as shown in Table 1.

**Table 1:** **Data used in three layer model simulations**.

**Description**	**Symbol**	**Value**
Total number of nodes	*N*	500
Subgroup of connected nodes	*n*	100
Size of injury	*m*	Depends on the case
Middle layer threshold	θ_*ml*_	5.54
Output layer threshold	θ_*output*_	5.54
Noise amplitude	β	0.25
Hebbian learn parameter a	*a*	1.5
Hebbian learn parameter b	*b*	0.5
Hebbian learn parameter c	*c*	0.5
Maximum connection weight	*w*_*max*_	10.3
Minimum connection weight	*w*_*min*_	0.0
Base learning rate	η_0_	0.01
Beginning of the learning rate drop	*H*_0_	5
End of the learning rate drop	*H*_*c*_	Depends on the case

In the pre-lesion phase, we start with random binary patterns with 50% active nodes in the input and output layer and give enough time for the system to learn. In the lesion phase, we kill a group of neighbor nodes immediately or gradually in the middle layer and wait for recovery of the system. The fraction $\frac{n}{N}$ measures network completeness in the sense that if we remove a group of *m* neighbor nodes in the middle layer and *m* > *n*, the route from input layer to output layer for some nodes will be cut. For *m* < *n*, although the network may be initially disabled, it has the potential to recover itself. In each trial one input is shown to the first layer and the connection weights are changed according to a Hebbian-like learning rule, Equation (2), as long as a difference exists between the desired and the actual output. Also at each time step we calculate Hamming distance (*H*) in the output layer as a measure to quantify network disability.

We consider three different types of lesions: immediate, gradual, and resection. For immediate injuries, we remove a total number of *m* nodes, simultaneously. In the gradual injury, we remove adjacent nodes one by one in specific time steps, until the total number of *m* nodes (defect size) are removed. The time interval between these removal time steps is quantified with a parameter called inter-removal time (*IRT*). Resection injury (Stewart and Ades, 1951; Meyer et al., 1958; Adametz, 1959; Finger et al., 1971; Rosen et al., 1971; Glick and Zimmerberg, 1972; Patrissi and Stein, 1975), is an intermediate between immediate and gradual, in which the *m* nodes, considered to be removed, are divided in *n*_*p*_ packages, and packages (each containing $m_{p}=\frac{m}{n_{p}}$ nodes) are removed, as a whole, every *IRT* time units. The immediate injury can be considered as either the second or the third type with *IRT* = 0.

### 2.2. Discussion on the learning rate, η

In the simplest case, the learning rate, η, introduced in Section 2, can be assumed constant. However, to be more realistic η should be a function of the network disability. We define a modified learning rate as a tool for better interpretation of the results of dynamic changes based on the network damage and disability.

Here, we assume a worst case scenario, in which the learning rate is a decreasing function of network disability. The aim is to show that although the network declines in performance due to a lower learning rate (note that we don't intend to improve the performance relative to when the rate is held fixed) the system still does better when damage is gradual compared to a sudden damage. It is known, for example, that many traumatic injuries, such as a sudden blow to the head, result in anterograde or retrograde amnesia. The mechanism, although not yet known, involves some disruption of synaptic plasticity effecting both memory and learning processes. We may interpret this, however, as a decrease in learning rate which also depends on both the spatial and temporal extend of the damage.

Intuitively, one expects that after a serious injury which yields a major disability, the network fails in recovery, so there should be a critical size of disability, *H*_*c*_, above which the system may not be recovered (the learning rate, η ~ 0). On the other hand, when *H* < *H*_0_, the learning rate more or less has a constant value (η ~ η_0_). Here in our model, we let η decrease smoothly with the size of the network disability from η_0_ to 0. The behavior of the system is not sensitive to the exact relation of η and *H*, but it is sensitive to the critical value of disability, *H*_*c*_. η(*H*) is defined as

(4) η={η0if 0≤H<H0(Fixed η for small H)η0 (1−(H−H0Hc−H0)2)12if H0≤H<Hc(η decreasesslowly at first andrapidly later)~0if Hc≤H(η~ 0 for large H)·

**Figure 2:**
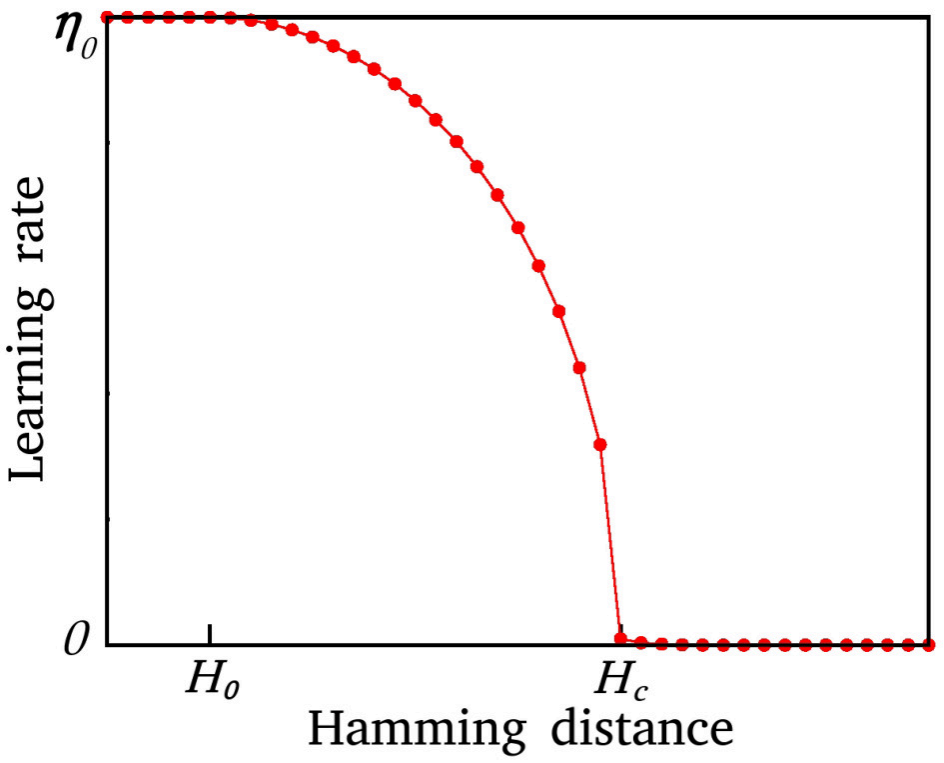
**The modified η as a function of network disability, *H***. η is constant for *H* ≤ *H*_0_, it decreases smoothly for *H*_0_ < *H* ≤ *H*_*c*_, and is zero for *H* > 0. A system with disability higher than a critical value can be rarely recovered.

Figure 2 shows the shape of the modified η. In the homeostasis model described in Section 3 the learning rate is also a function of network disability, but an increasing one.

### 2.3. Three layer model results

**Figure 3:**
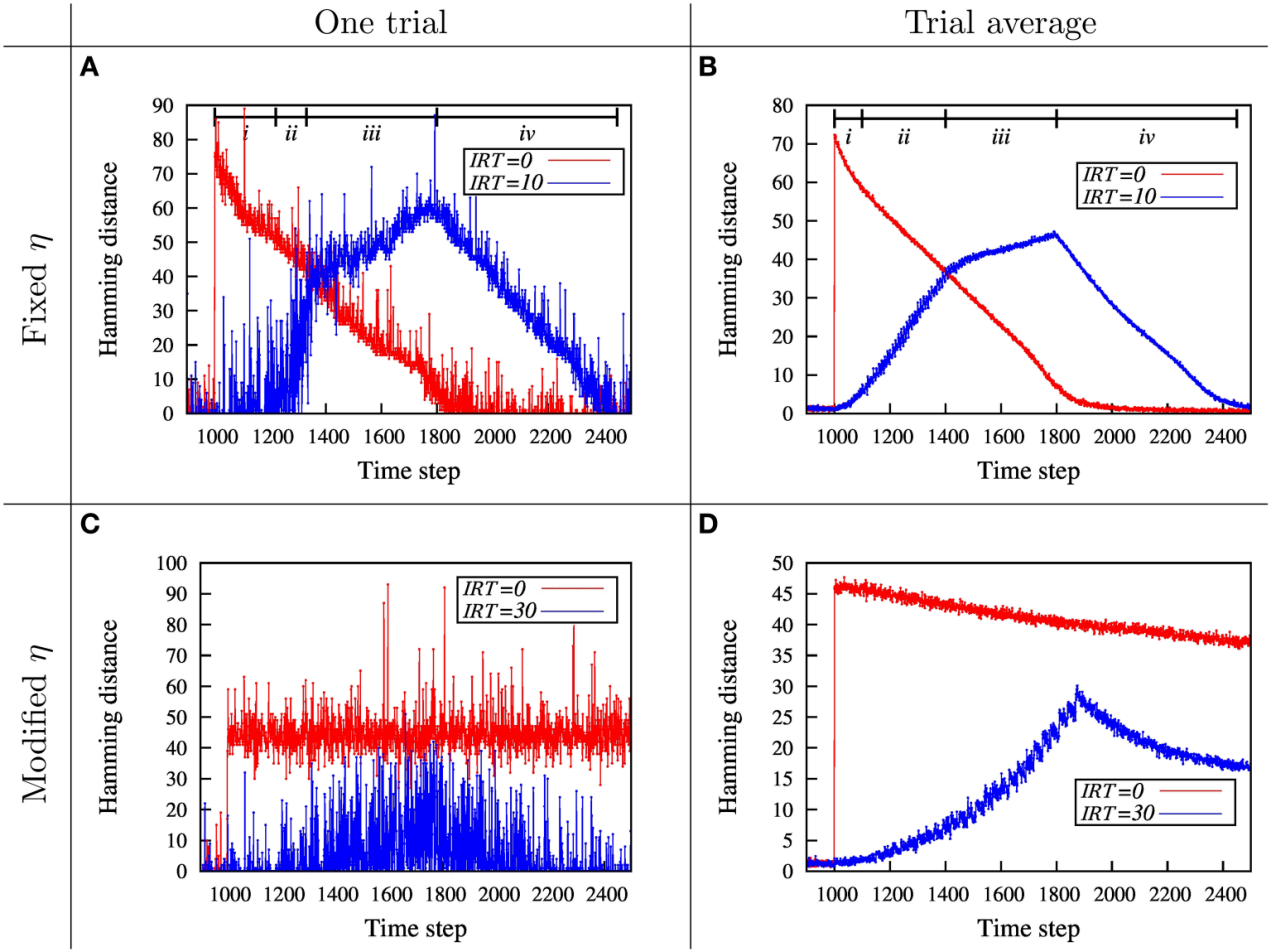
**Dynamics of network disability, ***H***, for total removal of ***m*** = 80 neighboring nodes for fixed and modified η. (A)** The dynamics of the network disability, measured by Hamming distance, is shown for immediate and gradual injury. When the nodes are removed suddenly (red curve), MAoD is higher compared to the gradual removal of the nodes (blue curve). During the removal of the first few nodes (The region shown by i), the network resists damage and shows a small disability. Thereafter, the hamming distance suddenly increases and the network shows a severe disability (region *ii*). Removing more nodes from time step 1300 to 1800 (region *iii*) which corresponds to removing 30–80th nodes, doesn't change network disability so much. After removing the last node, network starts to recover, roughly linearly, like the red curve (*iv*). The existence of a critical injury size could be inferred from the blue curve. **(B)** All the the parameters in this diagram are equal to parameters in **(A)**, but this one is the average of 200 different runs. The behavior in the lesion phase is the same as in **(A)**. **(C)** Simulation with modified η for the injury size of *m* = 30 for a single simulation. Red and blue curves correspond to *IRT* = 0 and *IRT* = 30, respectively. **(D)** Simulation with modified η. For this curves we take the average of 200 runs with different initial conditions. The parameters are the same as in **(C)**. *H*_0_ and *H*_*c*_ of modified η are 5 and 35, respectively, in **(C,D)**. Effect of *H*_*c*_ can be observed by comparing **(C)** with the corresponding plot in Supplementary Figure S2.

**Figure 4:**
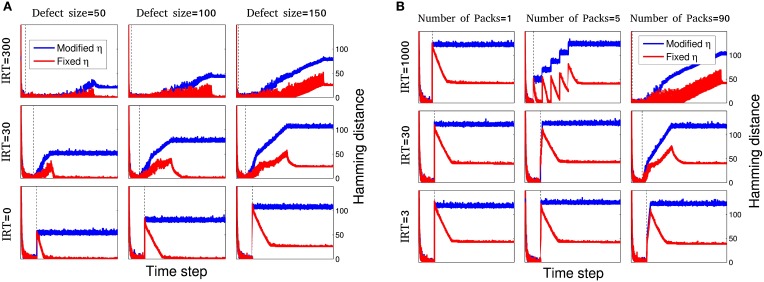
**Dynamics of network disability for different spacio-temporal damages and learning procedures**. For the maximum amount of *m* = 180 nodes and inter-removal time, in the range of *IRT* = 0 (immediate injury) to *IRT* = 1000, different systems are simulated for 9 different initializations. A small subset of these simulations are shown here. **(A)** For gradual injury, the effect of defect size, *m*, and inter-removal time, *IRT*, is shown. For each case, dynamics of network disability is shown for fixed (red) and modified η (blue). For larger inter-removal times, even for large injury sizes the network shows relatively small disability. Note that the blue curves show a higher disability and stay above the red curves due to a decreasing learning rate but the blue curves tend to lower values of MAoD as *IRT* is increased as in the case of fixed learning, going from bottom to top in the middle column for example. Time ranges are different for different plots. To make the plots comparable, vertical dashed line in each sub plot shows time step *t* = 1000 which is the injury start time. **(B)** For resection injury, the effect of number of packages, *n*_*p*_ and inter-removal time, *IRT*, is studied. For each case of package number and inter-removal time, dynamics of network disability is shown for fixed (red) and modified η (blue). Increasing the number of packages and *IRT*, decreases the MAoD. Here, *H*_*c*_ = 25.

To study the relation between damage size and temporal pattern of injury, we consider the dynamics of network disability for fixed and modified η in Section 2.3.1. Figure 3 shows a basic trend for a fixed pattern of injury, but different temporal pattern of damage. In Section 2.3.2 this relation is shown for different patterns of injury. Here, we rely on a more useful and informative parameter which we call maximum amount of disability (MAoD) which is the maximum disability the system endures during the simulation after the damage starts. This, rather than the ultimate disability of the system, is used because it is more reliable while in general higher MAoD decreases the chance of recovery. Ultimate behavior of systems with modified η can best show this relation between MAoD and recovery rate as illustrated in Figure 4.

#### 2.3.1. Three layer model—the effect of learning rate

***Fixed* η**

The results for the immediate and gradual injury is shown in Figure 3A. In both cases the total injury size is the same. This diagram shows how damage, as quantified by the Hamming distance, evolves with time. Here the recovery/learning rate, η, is a constant function of disability size. Note that the nodes are removed after a pre-learning period.

In the case of immediate injury, a roughly linear recovery over time is observed. In the gradual injury case, a complex dynamics is observed. At first, there is a period in which the network resists the injury, but thereafter suddenly suffers a huge disability, which implies a possible critical size of injury. After this sudden increase of disability which implies a possible network break down, disability still increases until the time the last node is removed, followed by a linear recovery similar to the case of immediate injury.

To show that these results not just hold for a specific initialization, we simulated the three layer model with the same parameters for many different initializations. In Figure 3B the average of all these simulations is shown. It can be seen that the previous effect as in Figure 3A still holds. Note that the MAoD is smaller in gradual injuries than in the sudden injuries.

***Modified* η**

To better observe the effect of critical injury size, we use the modified relation between the learning rate η, and disability *H*, described in Section 2.2 (Also, see Figure 2). This dependency of η on *H* captures the state of severe disability. For hamming distances larger than *H*_*c*_ the learning rate, η, is close to zero and therefore no learning is observed. Figure 3C shows two single runs, for the immediate and the gradual injury with modified, damage dependant, η. When the injury is immediate, MAoD becomes larger than *H*_*c*_ so the recovery rate η drops down suddenly and the system can not be recovered. In gradual injury the Hamming distance doesn't reach the value which gives η ~ 0, hence the system can recover. In Figure 3D the same results for the average of many different initializations is shown. The effect of *H*_*c*_ can be observed by comparing Figure 3C and the corresponding plot in Supplementary Figure S2 where *H*_*c*_ is 35 and 25, respectively. While the system has the chance to recover with larger *H*_*c*_, it will endure high value of MAoD with smaller *H*_*c*_.

#### 2.3.2. Three layer model—the effect of injury types and recovery models

In the previous section the behavior of three layer model for immediate and gradual injury has been shown briefly. Here according to injury and recovery models four kind of sub-models is studied:

Type 1: Gradual injury with fixed η.

Type 2: Gradual injury with modified η.

Type 3: Resection injury with fixed η.

Type 4: Resection injury with modified η.

In each sub-model the relation between size and temporal pattern of injury, is studied.

***Type 1: Gradual injury—Fixed* η**

For gradual injury the effect of defect size, *m*, and inter-removal time, *IRT*, is shown in Figure 4A. Here, the dynamics of network disability for fixed and modified learning rate can be compared for different cases of damage size and *IRT* of network nodes. The result shows that despite larger injury size, the network may endure a smaller disability with larger *IRT*. Here, the same result, i.e., gradual injury helps network recovery, is obtained for all the cases considered.

**Figure 5:**
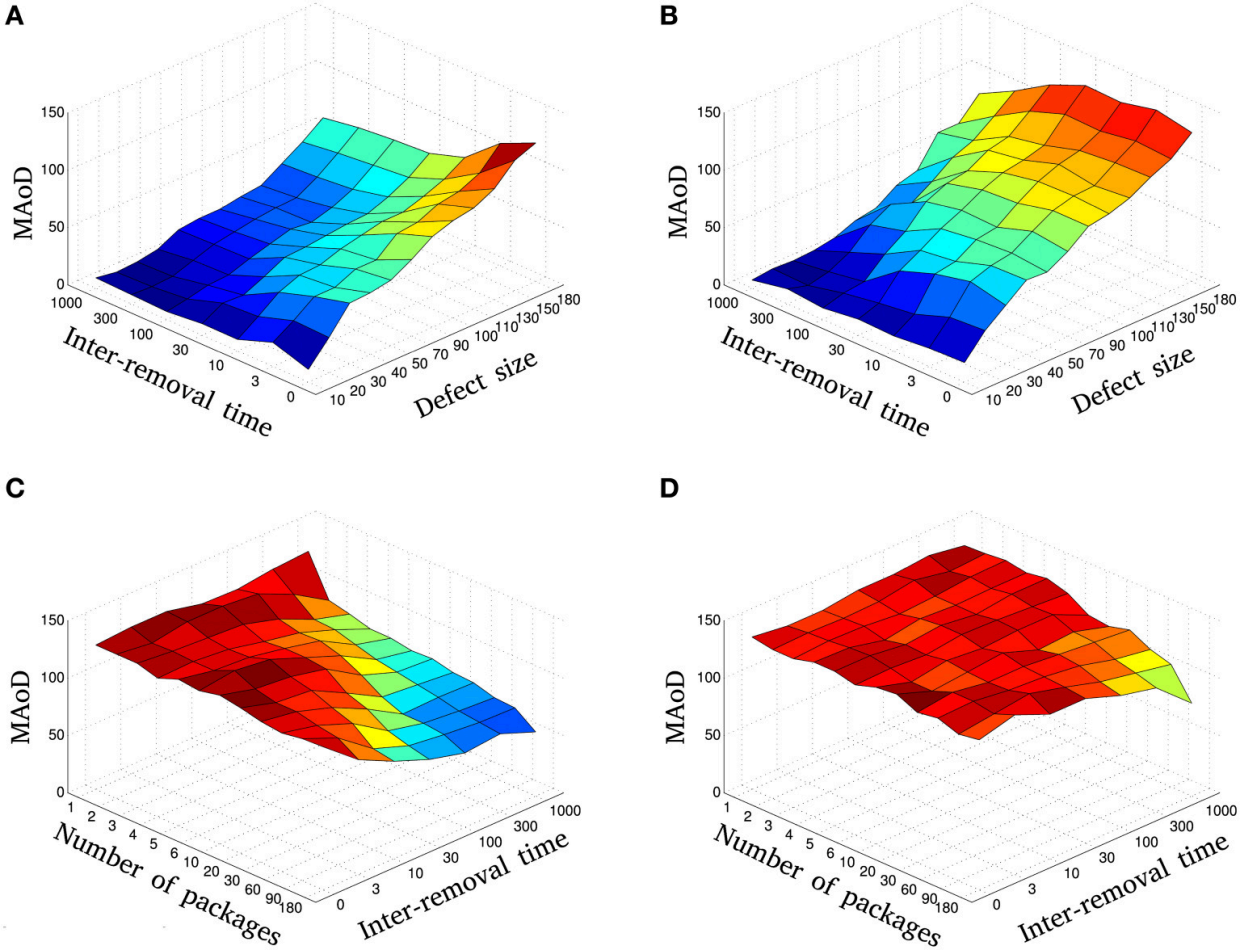
**The relation between size and temporal pattern of injury: The MAoD of gradual injury is plotted vs. the different injury sizes, ***m***, and inter-removal times, ***IRT***, using fixed (A) and modified η with ***H***_***c***_ = 25 (B)**. Blue to red represent low to high MAoD, respectively. Compare, for instance, MAoD values in **(A)** for *IRT* = 0 and 70 ≤ defect size < 180 with the values for *IRT* ≥ 10 and defect size = 180. Also, note that whilst in Figure 5B there is an overall decline in performance, compared to fixed learning rate, the MAoD tends to decrease as a function of inter-removal time for all defect sizes. The MAoD of resection injury is plotted vs. the different number of packages, *n*_*p*_, and inter-removal times, *IRT*, using fixed **(C)** and modified η **(D)**. Blue to red represent low to high MAoD, respectively. Diagrams **(A,B)** show that with fast and small injury we can have the same MAoD (same color in the diagrams) as we would have with slow but large injuries. The same holds for fine removal of nodes (more packages) but fast vs. coarse removal (less packages) but slow (see **C,D**). These diagrams are the average of 9 independent simulations with different initial conditions.

The MAoD is estimated and summarized in Figure 5A. The relation between damage size and temporal pattern of injury is shown in this figure. One sees that a system with large injury and *IRT* could have smaller MAoD than a system with smaller injury and *IRT*. This observation supports our claim that a rapid small damage could be more harmful than a bigger but slower one.

Although Figure 5A represents the overall relation between the damage size and the *IRT*, complete dynamics for different values of these parameters can be seen in Supplementary Figure S1.

***Type 2: Gradual injury—Modified* η**

This section is similar to the previous section except for the learning rate η. Here, we use a modified η as described in Section 2.2. Figure 4A shows the behavior of this sub-model for some selected simulations. Note that the disability stays higher with the damage-dependent model due to a decreasing learning rate, and MAoD decreases by increasing *IRT*.

Looking at Figure 5B, we first note that there is an overall decline in performance compared to when the learning rate is held fixed (increase in MAoD). MAoD is about the same in the regions with defect size less than *H*_*c*_ for fixed and modified η, as expected (Figures 5A,B), and again, the MAoD tends to decrease as a function of *IRT*. As seen again, a system with large injury and large *IRT* could have smaller MAoD than a system with small injury and small *IRT*.

Complete set of simulations can be seen in Supplementary Figure S2.

***Type 3: Resection injury—Fixed* η**

Based on the findings and experiments of previous studies, Stewart and Ades (1951), Meyer et al. (1958), Adametz (1959), Finger et al. (1971), Rosen et al. (1971), Glick and Zimmerberg (1972), and Patrissi and Stein (1975) we know that MAoD in a resection injury is less than an immediate injury.

To study this kind of injury in our model, we set the injury size to *m* and divided them to *n*_*p*_ packages, each contains $m_{p}=\frac{m}{n_{p}}$ neighboring nodes. Packages are sequentially removed with time interval equal to *IRT*.

Simulations were done for different values of *n*_*p*_ and *IRT*. In Figure 4B a selection of these simulations is shown in red. In agreement with the result of previous sections, more packages and larger *IRT*, decrease MAoD although the total injury size is fixed, i.e., gradual injury reduces MAoD. Same as the previous sections, the MAoD is plotted in Figure 5C which shows the effect of package numbers and *IRT* on MAoD. Removing more packages with smaller *IRT* has the same effect on MAoD as removing less packages but with higher *IRT*. Complete set of simulations can be seen in Supplementary Figure S3.

***Type 4: Resection injury—Modified* η**

For a fixed injury size and modified η described in Section 2.2, different systems are simulated for various *IRT*s and *n*_*p*_s. In Figure 4B a selection of these simulations is shown. The MAoD is plotted in Figure 5D. Note that although the total number of removed nodes is much greater than *H*_*c*_ and *n*, subgroup of connected nodes explained in Section 2.1, still the same behavior as seen in the previous section is visible for high number of packages and high *IRT*. Complete set of simulations can be seen in Supplementary Figure S4.

## 3. Homeostasis model

### 3.1. The model

**Figure 6:**
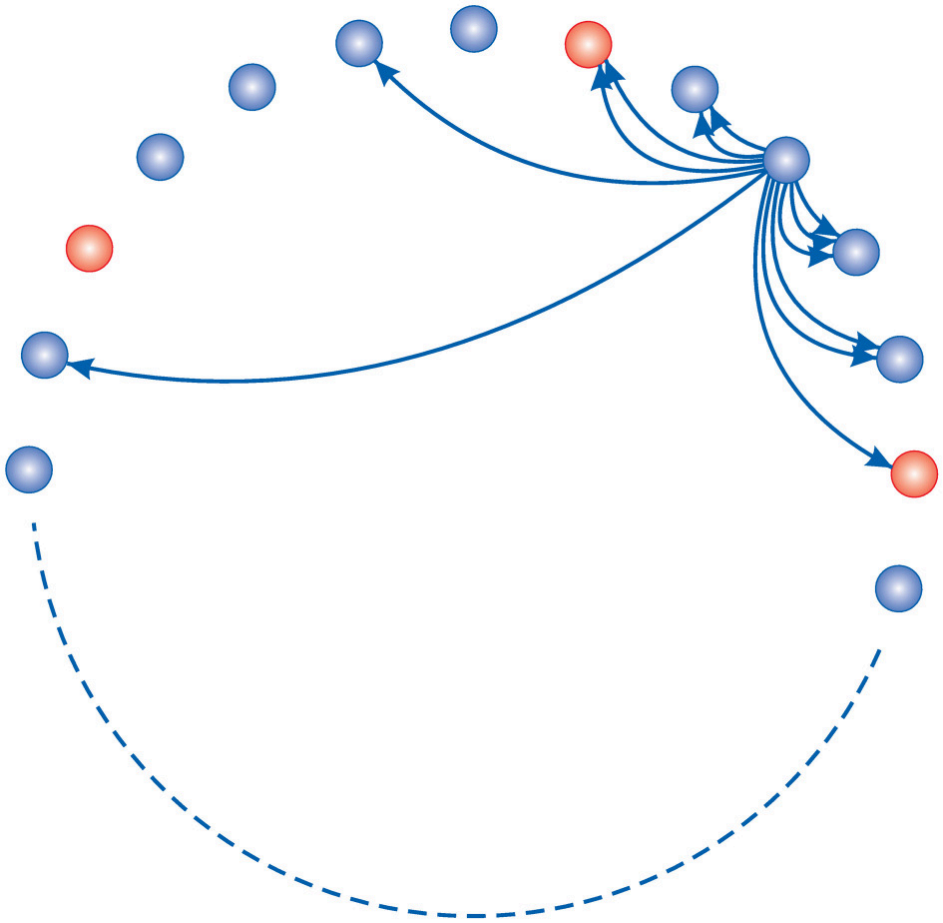
**Homeostasis model network topology: 80% of neurons (blue) are excitatory and the rest of them are inhibitory (red)**. Each neuron has connections to its neighbors. They can make new connections with nearer neighbors easier. In this study there are a total of *N* = 100 neurons.

The feed-forward model introduced in the previous section is not a very realistic model for cortical circuits, where neurons are recurrently connected. In this section we use a more realistic model based on a recurrent network, initially proposed by Butz (Butz et al., 2008, 2009), and compare it with the result of our three layer model in the previous section. For details of the homeostasis model see (Butz et al., 2008). The model consists of 100 simple spiking neurons on a ring where 80% of them are excitatory and the rest are inhibitory (Figure 6). The activation function for neurons in this model is:

(5) $$\begin{array}{l} F\left(X_{i}^{t}\right)=\frac{1}{1+e^{-\beta \left(X_{i}^{t}-\theta \right)}} \end{array}$$

where θ is firing threshold and β defines the steepness of the sigmoid function and determines the noise amplitude. *X*_*i*_ is a summation over all excitatory and inhibitory connections to neuron *i* plus an external input obtained from a Poisson distribution which plays the role of an external noisy input. For more information on network topology see (Butz et al., 2008, 2009).

When the internal conditions of a dynamic system remain stable and relatively constant during regulation of its variables, the system is in homeostatic state. In neuroscience context, the state of a network where the firing rate of neurons are similar to a moderate firing rate is here called a homeostatic state. It has been shown (Mattson et al., 1988; Lipton and Kater, 1989) that for biological networks there is a moderate firing rate that may be different for each network. In this model assuming that there is a homeostatic state, the firing rate or the probability of firing in each time step is set to 0.5.

In each time step, we update the neuronal activity but the number of connections does not change. After each 100 time steps, which is called a morphological time step, according to neuronal activity in the last 1000 time steps, we change the number of connections to reach homeostasis. Average over the last 1000 time steps is used to prevent short time fluctuations and unrealistic changes in the number of connections. In each time step first *X*_*i*_, the input of each neuron *i*, is calculated. Then we update the firing probability, *F*, of each neuron with a sigmoid activation function. After updating *F*, we update the state of each neuron which fires with probability *F*. In each morphological time step, we change the number of connections according to the history of *F*. To reach homeostasis, we demand that the average of *F* over last 1000 time steps converges to 0.5. For the average firing rate greater than 0.5, we change the number of connections to decrease it. We do the opposite for neurons with the average of *F* smaller than 0.5. For input connections, first we calculate

(6) $$\begin{array}{l} \Delta I_{i}=\nu \cdot \Delta \bar{F_{i}}\cdot I_{i} \end{array}$$

where ν is a small number that adjusts the rate of convergence to homeostasis, $\Delta \bar{F_{i}}=\bar{F_{i}}-0.5$ and *I*_*i*_ is the number of input connections of neuron *i*. Changing the number of output connections is similar to this relation.

In Equation (6), the rate of change is proportional to $\Delta \bar{F_{i}}$, which shows how far the neuron is from homeostasis, or, how bad the neuron works. Taking the variance of *F* over all neurons, we obtain a parameter showing how far the whole network is from homeostasis.

In summary, supposing that the variance of *F* over all nodes in past 1000 time steps is what may be called network disability, we aim to study the relation between temporal pattern of injury and network disability in this model. To study this, first we let the network reach homeostasis, then we start killing the neurons by disconnecting them from the other network nodes. For the number of nodes being removed (injury size, *m*, in previous section), we remove them all at once (immediate injury) or gradually one by one every *IRT* morphological time steps (gradual injury). All the constants are set as shown in Table 2. The values used in this study are borrowed from Butz et al. (2008, 2009) in which more information about the choice of parameters and more details about homeostasis model can be found.

**Table 2:** **Data used in the homeostasis model simulations**.

**Description**	**Symbol**	**Value**
Total number of nodes	*n*_*nodes*_	100
Morphological time step	*T*	100
Threshold	θ	500
Noise amplitude	β	0.002
Probability of deletion	*p*_*del*_	0.1
Learning rate	ν	0.005
External input rate	λ	30

### 3.2. Homeostasis model results

**Figure 7:**
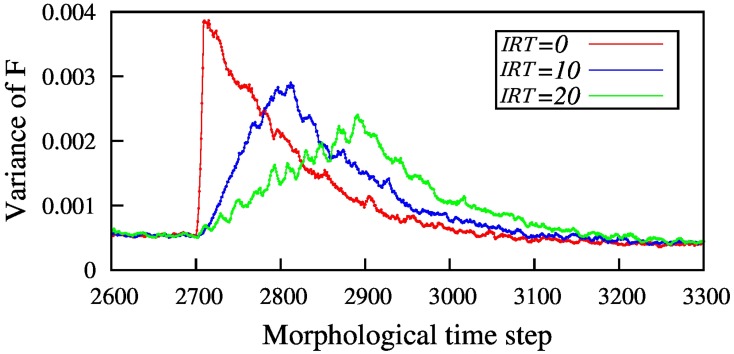
**The amount of network disability (shown by variance of ***F***) for different inter-removal time, for ***N*** = 100 nodes and removing 10 neighboring nodes**. By increasing the inter-removal time, *IRT*, the maximum disability of the network is decreased. Here, red line shows immediate injury but blue and green ones show gradual injury with 10 and 20 time steps between node removal, respectively. Injury starts for all the simulations at *T* = 2700 so that the network has enough time to reach homeostasis before starting the injury.

In Figure 7 the result of homeostasis model for a fixed defect size and some *IRTs* is shown. This figure shows how the network disability, quantified by the variance of *F*, evolves with time for different *IRT*s. In the simulations, the network is let to reach homeostasis before the injury starts. As seen in the Figure 7 the amount of network disability declines as *IRT* increases. This result is in agreement with the results in the three layer model.

One important point about homeostasis model is that the recovery rate is proportional to the size of disability, in contrast with the three layer model with modified η. This can explain why all three different injury patterns recover almost at the same time.

## 4. Discussion

Using two simple network models, we studied the effect of temporal pattern of node destruction on network recovery. In the three-layer model the learning rate is either a constant or a decreasing function of overall disability. In homeostasis model, the learning rate which leads to recovery is an increasing function of system disability. Regardless of how the the learning rate, η, changes, increasing the time interval between removing the nodes, *IRT*, leads to a decrease in the maximum amount of network disability (MAoD). In the case of our simple three layer model the MAoD is much higher when nodes in the process layer are removed at once (Figure 3). By removing the nodes gradually MAoD is reduced, as shown in Figures 5A,B. The same results hold for the more biologically inspired model, the homeostasis model (Figure 7). In both cases big sudden injury results in serious disability but large time intervals between removing nodes decreases MAoD effectively. In general, these results show a generic property of a distributed network such that when some nodes are removed gradually the remaining nodes take part in the process of recovery. More complex interactions, however, may also be involved explaining the difference of a sudden vs. gradual damage.

It is reported that following damage, there is a time period in which the process of recovery speeds up (Duffau et al., 2003). Also, there are some animal models showing that a focal damage induces excitability and plasticity in the rest of the brain (Buchkremer-Ratzmann et al., 1996). So, while we base our three-layer network modeling on the more plausible assumption that the rate of learning deceases as a function of network damage for big damages, the reports stated above show clearly that many unknowns about the process of brain recovery are yet to be discovered. One may allude to many experimental findings reported in the literature on the role of neurogenesis following damage to the hippocampus, for example. What is important in our case based on the modeling result is that regardless of changes of learning rate, η, for both networks increasing the time interval between removing the nodes, *IRT*, leads to a decrease in the maximum amount of network disability (MAoD). More experimental findings about the relation between learning rate and network disability may lead to more realistic results. This is why, in the absence of such findings, we only study MAoD instead of the long-run behavior of the system. Although the origins and mechanisms of these changes are still unknown it is worth studying these models in a more realistic setting, for example, it is known for a quite a while that brain recovery after sequential lesions depends on the amount of tissue resected at each surgical stage (Stein et al., 1977). In similar animal studies it is shown that in general a two-stage lesion has a much better chance of recovery than a one stage acute lesion (Finger et al., 1971). These are clear examples of what may be called a spatio-temporal pattern of brain damage. More importantly as has been argued by Daffauand et al. (Duffau et al., 2003) the same sort of mechanism may be at work in aging where up to a certain threshold the system can no longer cope with the neural destruction. This indeed would be an interesting future research requiring a more realistic neural modeling.

### Conflict of interest statement

The authors declare that the research was conducted in the absence of any commercial or financial relationships that could be construed as a potential conflict of interest.

## References

[B1] Adametz J. H. (1959). Rate of recovery of functioning in cats with rostral reticular lesions; an experimental study. J. Neurosurg. 16, 85–97. discussion: 97–98. 13621267 10.3171/jns.1959.16.1.0085

[B2] Albert R. Jeong H. Barabási A. L. (2000). Error and attack tolerance of complex networks. Nature 406, 378–382. 10.1038/35019019 10935628

[B3] Alstott J. Breakspear M. Hagmann P. Cammoun L. Sporns O. (2009). Modeling the impact of lesions in the human brain. PLoS Comput. Biol. 5:e1000408. 10.1371/journal.pcbi.1000408 19521503 PMC2688028

[B4] Amit D. J. (1992). Modelling Brain Function: The World of Attractor Neural Networks, 1st Edn. New York, NY: Cambridge University Press.

[B5] Bondarenko V. E. (2005). Information processing, memories, and synchronization in chaotic neural network with the time delay. Complexity 11, 39–52. 10.1002/cplx.20103

[B6] Buchkremer-Ratzmann I. August M. Hagemann G. Witte O. W. (1996). Electrophysiological transcortical diaschisis after cortical photothrombosis in rat brain. Stroke 27, 1105–1109. discussion: 1109–1111. 8650722 10.1161/01.str.27.6.1105

[B7] Butz M. Teuchert-Noodt G. Grafen K. van Ooyen A. (2008). Inverse relationship between adult hippocampal cell proliferation and synaptic rewiring in the dentate gyrus. Hippocampus 18, 879–898. 10.1002/hipo.20445 18481284

[B8] Butz M. van Ooyen A. Wörgötter F. (2009). A model for cortical rewiring following deafferentation and focal stroke. Front. Comput. Neurosci. 3:10. 10.3389/neuro.10.010.2009 19680468 PMC2726035

[B9] Calautti C. Baron J.-C. (2003). Functional neuroimaging studies of motor recovery after stroke in adults: a review. Stroke 34, 1553–1566. 10.1161/01.STR.0000071761.36075.A6 12738893

[B10] Callaway D. S. Newman M. E. J. Strogatz S. H. Watts D. J. (2000). Network robustness and fragility: percolation on random graphs. Phys. Rev. Lett. 85:4. 10.1103/PhysRevLett.85.5468 11136023

[B11] Cohen R. Erez K. Ben-Avraham D. Havlin S. (2000a). Breakdown of the internet under intentional attack. Phys. Rev. Lett. 86:4. 10.1103/PhysRevLett.86.3682 11328053

[B12] Cohen G. Johnston R. A. Plunkett K. (2000b). Exploring cognition: Damaged brains and neural networks: readings in cognitive neuropsychology and connnectionist modelling Hove, UK: Psychology Press.

[B13] Desmurget M. Bonnetblanc F. Duffau H. (2007). Contrasting acute and slow-growing lesions: a new door to brain plasticity. Brain 130(Pt 4), 898–914. 10.1093/brain/awl300 17121742

[B14] Duffau H. Capelle L. Denvil D. Sichez N. Gatignol P. Taillandier L.. (2003). Usefulness of intraoperative electrical subcortical mapping during surgery for low-grade gliomas located within eloquent brain regions: functional results in a consecutive series of 103 patients. J. Neurosurg. 98, 764–778. 10.3171/jns.2003.98.4.0764 12691401

[B15] Duffau H. Denvil D. Capelle L. (2002). Long term reshaping of language, sensory, and motor maps after glioma resection: a new parameter to integrate in the surgical strategy. J. Neurol. Neurosurg. Psychiatry 72, 511–516. 10.1136/jnnp.72.4.511 11909913 PMC1737830

[B16] Finger S. Marshak R. A. Cohen M. Scheff S. Trace R. Niemand D. (1971). Effects of successive and simultaneous lesions of somatosensory cortex on tactile discrimination in the rat. J. Compar. Physiol. Psychol. 77, 21–227. 5117202 10.1037/h0031648

[B17] Fortney K. Pahle J. Delgado J. Obernosterer G. Shah V. Wojnowicz M. (2007). Effects of simulated brain damage on small-world neural networks, in Proceedings of the Santa Fe Institute Complex Systems Summer School (Santa Fe, NM).

[B18] Glick S. D. Zimmerberg B. (1972). Comparative recovery following simultaneous- and successive-stage frontal brain damage in mice. J. Compar. Physiol. Psychol. 79, 481–487. 4341017 10.1037/h0032814

[B19] Goldman M. S. (2009). Memory without feedback in a neural network. Neuron 61, 621–634. 10.1016/j.neuron.2008.12.012 19249281 PMC2674525

[B20] Lee H. Lee D. S. Kang H. Kim B.-N. Chung M. K. (2011). Sparse brain network recovery under compressed sensing. IEEE Trans. Med. Imaging 30, 1154–1165. 10.1109/TMI.2011.2140380 21478072

[B21] Lipton S. A. Kater S. B. (1989). Neurotransmitter regulation of neuronal outgrowth, plasticity and survival. Trends Neurosci. 12, 265–270. 2475939 10.1016/0166-2236(89)90026-x

[B22] Mattson M. P. Taylor-Hunter A. Kater S. B. (1988). Neurite outgrowth in individual neurons of a neuronal population is differentially regulated by calcium and cyclic AMP. J. Neurosci. 8, 1704–1711. 2835450 10.1523/JNEUROSCI.08-05-01704.1988 PMC6569213

[B23] Meyer D. R. Isaac W. Maher B. (1958). The role of stimulation in spontaneous reorganization of visual habits. J. Compar. Physiol. Psychol. 51, 546–548. 13587681 10.1037/h0043002

[B24] Murre J. M. J. Griffioen R. Robertson I. H. (2003). Selfreparing neural networks: a model for recovery from brain damage, in Knowledge-Based Intelligent Information and Engineering Systems, Vol. 2774, eds Palade V. Howlett R. J. Jain L. (Berlin: Springer), 1164–1171.

[B25] Nawrocki R. A. Voyles R. M. (2011). Artificial neural network performance degradation under network damage: stuck-at faults, in The 2011 International Joint Conference on Neural Networks, Vol. 1 (San Jose, CA: IEEE), 442–449.

[B26] Pantano P. Formisano R. Ricci M. Piero V. Sabatini U. Pofi B.. (1996). Motor recovery after stroke: Morphological and functional brain alterations. Brain 119, 1849–1857. 10.1093/brain/119.6.1849 9009992

[B27] Patrissi G. Stein D. G. (1975). Temporal factors in recovery of function after brain damage. Exp. Neurol. 47, 470–480. 1132460 10.1016/0014-4886(75)90079-5

[B28] Rosen J. Stein D. Butters N. (1971). Recovery of function after serial ablation of prefrontal cortex in the rhesus monkey. Science 173, 353–356. 4997798 10.1126/science.173.3994.353

[B29] Stein D. G. Butters N. Rosen J. (1977). A comparison of two- and four-stage ablations of sulcus principals on recovery of spatial performance in the rhesus monkey. Neuropsychologia 15, 179–182. 401532 10.1016/0028-3932(77)90128-2

[B30] Stewart J. W. Ades H. W. (1951). The time factor in reintegration of a learned habit lost after temporal lobe lesions in the monkey (*Macaca mulatta*). J. Compar. Physiol. Psychol. 44, 479–486. 14888743 10.1037/h0059803

[B31] Varona J. F. (2010). Long-term prognosis of ischemic stroke in young adults. Acta Neurol. Scand. 2011, 123–130. 10.4061/2011/879817 21197408 PMC3010699

[B32] Vazquez A. Moreno Y. (2002). Resilience to damage of graphs with degree correlations. Phys. Rev. E 67:4. 10.1103/PhysRevE.67.015101 12636544

[B33] Wang J. Qiao C. Yu H. Kong H. (2011). On progressive network recovery after a major disruption. Sci. Technol. 1, 1925–1933. 10.1109/infcom.2011.5934996

