# Supplementary figures and images for: Modeling spatio-temporal dynamics of network damage and network recovery
Source: Front Comput Neurosci. 2015 Oct 22;9:130. doi: 10.3389/fncom.2015.00130 (PMC4614320; doi:10.3389/fncom.2015.00130)

Type 1: No packages – Fixed  $\eta$

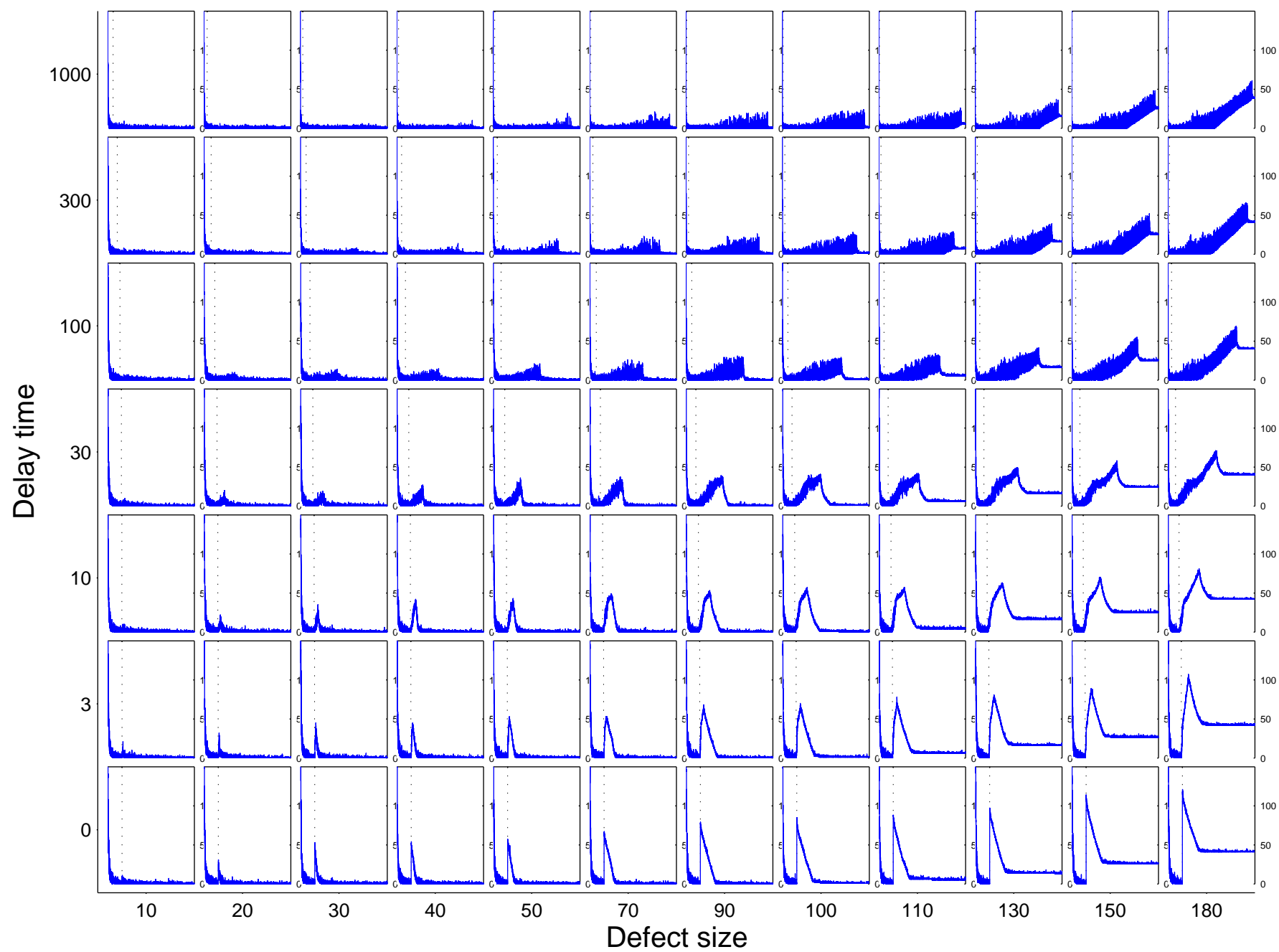

Supplement: Supplementary file 1 [file FigureS1.PDF]

Type 2: No packages – Modified  $\eta$

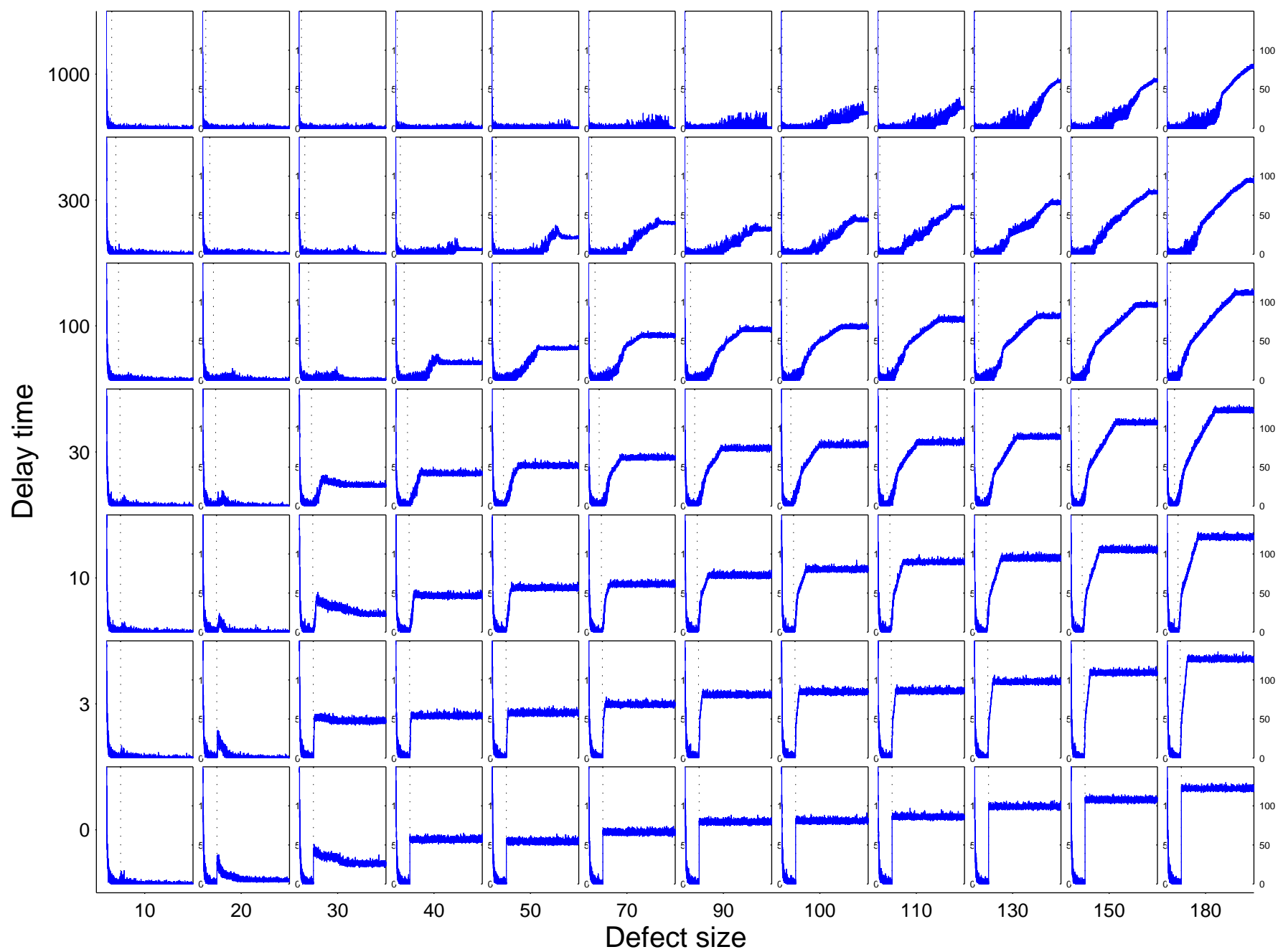

Supplement: Supplementary file 2 [file FigureS2.PDF]

Type 3: packages – Fixed  $\eta$

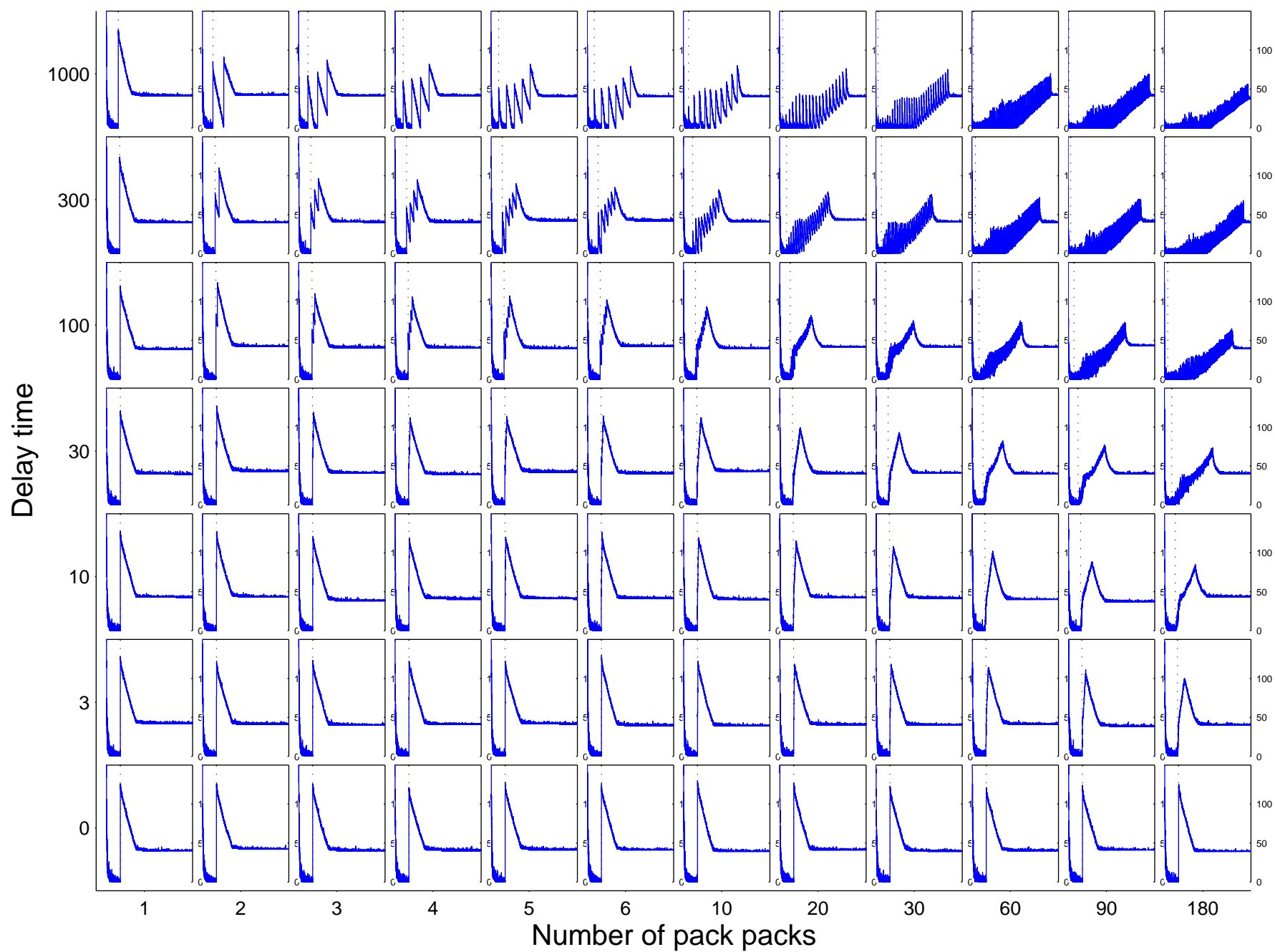

Supplement: Supplementary file 3 [file FigureS3.PDF]

## Type 4: packages – Modified $\eta$

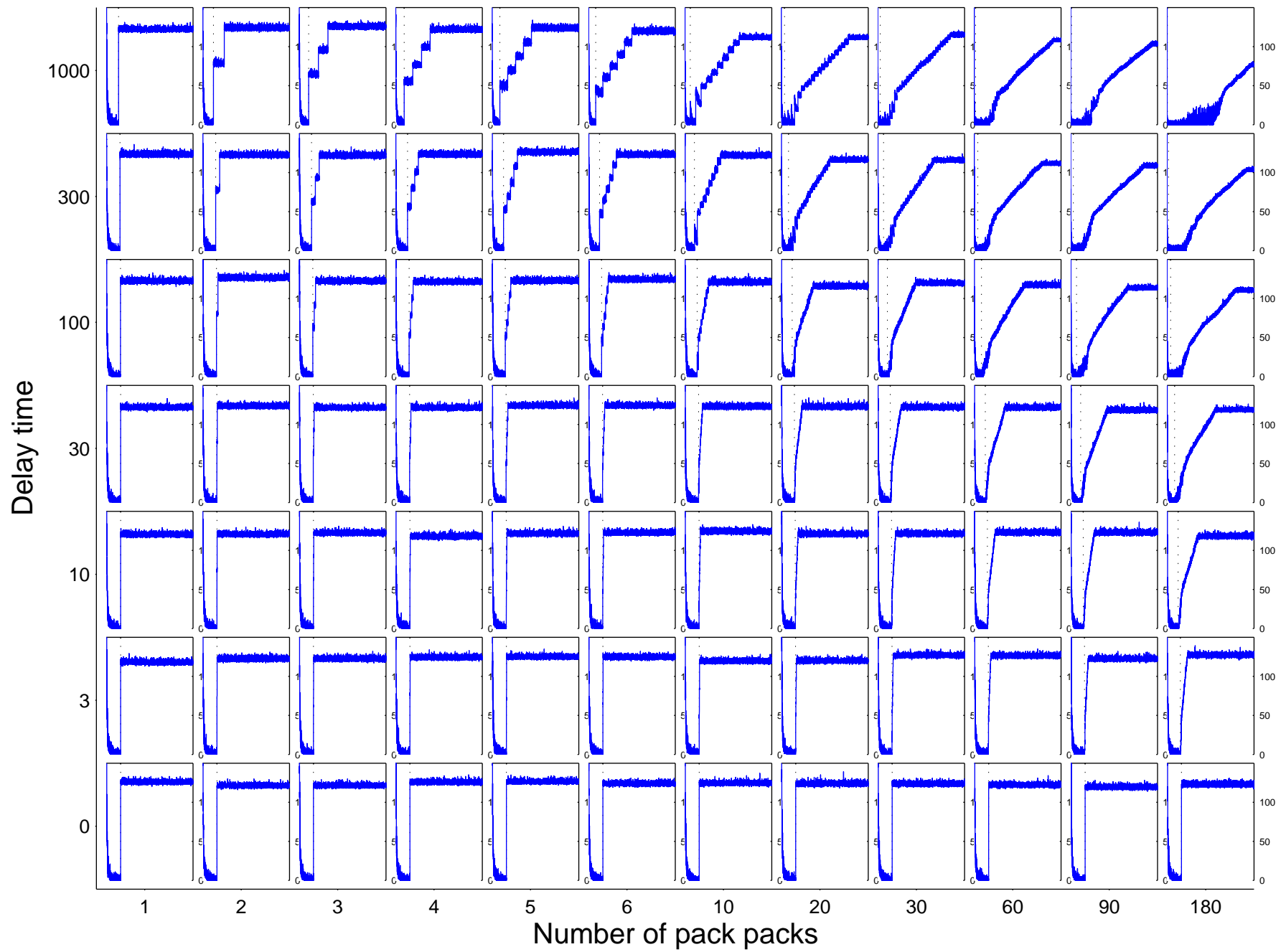

Supplement: Supplementary file 4 [file FigureS4.PDF]
